# Treatment of Dental Pulp

**Published:** 1859-02

**Authors:** 


### TREATMENT OF DENTAL PULP.

Mr. Editor:—If you think the following observations upon the treatment of the dental pulp, worthy a place in your very excellent "REPORTER," you can publish them; if not, you are at liberty to dispose of them as you see proper.

Whether the dental pulp, after exposure, can be preserved in a normal state, depends not only upon *its* condition of health, but also, very much upon the *bodily* condition of the patient. If the patient possesses a strumous diathesis, or is suffering with dyspepsia, or any intestinal derangement, the chances of success are very uncertain.

When I find the nerve and its investing membrane exposed, but in a healthy condition, and the dental arch of the patient fully developed, the gums healthy, and no predisposition to arterial excitement, I have no fears in regard to its successful treatment.

For a number of years it has been my practice to treat the exposed nerve, preparatory to filling, with a mixture of tannin and creosote. Latterly, I have been using glycerine in place of the creosote, and in many cases deem it preferable. In introducing the filling, the foil should be so packed, as not to press too hard immediately *over* the exposed nerve, (the pressure is to be feared;) at the same time the filling should be made solid, and *thoroughly* compact around its edges, where it joins the parietes of the cavity. In many cases where the dentine is very sensative, and will not admit of proper excavation, I use temporary fillings, which, after remaining in six months, I have removed, and invariably found that nature had been making efforts to protect herself against the encroachments of the disease, by consolidating the dental tubuli. I have very little faith in indiscriminate "nerve capping," although have used a horn cap in some few cases, where it was thought necessary.

But when the nerve is inflamed, and *these other conditions* unfavorable, I find it utterly futile to rely upon preservative treatment; but always destroy it, either with an instrument or an escharotic.

The former method is always preferable; but when the tooth and other circumstances will not admit of my using an instrument, I then use the following escharotic:

℞.—Morph. Sulph. xx grs.;
Arsen. Acid, xxx grs.;
Ole. Caryophyl., q. s., misce.

The oil of cloves is far more preferable than creosote, being less unpleasant and more agreeable to the olfactories of the patient. A small bit of this mixture of the size of a pin's head should be applied *in contact* with the exposed nerve, and the cavity filled with wax, and let remain for twenty-four hours. One application is generally sufficient. A proper opening should now be made into the cavity and all the decayed and dead pulp removed. Should there still be a tenderness in the fang or fangs, it is best to fill the cavity with cotton, moistened with spirits of camphor, and let the tooth rest for a few days. By this time the little remaining vitality of the nerve filaments will also be destroyed, when the balance of the nerve can be removed down to the apex of the root. After applying a solution of tannin in glycerine, and thoroughly washing, the roots and cavity are prepared for inserting the filling.

Should the operator neglect to remove all the pulp, but merely *wall it in* by the filling, it would be very apt to lead to inflammation of the alveolo-dental periosteum, terminating in alveolar abscess, and probably requiring the extraction of the tooth.

In the treatment of the dental pulp, like in all other operations in operative dentistry, it requires of the intelligent dentist, more than a mere *routine* system of therapeutics—he must be "eclectic," exercise his judgment, and use such remedies and means as the nature of the case demands.

Middletown, Md., Dec., 1858. J. P. H. B.

---

MR. EDITOR:—Having kindly solicited me to contribute a communication for the next number of the *Dental Reporter*, I herewith cheerfully comply with your request. Deeming the following cases not entirely void of interest, I transmitted them in *part* to the *Dental News Letter*, (if my memory be not at fault,) in the early part of 1855. But unfortunately quite a number of errors crept into the publication. The fault, however, in all probability, was my own, as I carelessly wrote the article in pencil.

If you coincide with me in regard to their worth, then give them publicity; if not, consign them to the flames. At some subsequent period, you may hear from me again, relative to some inventions, improvements, etc., which I have effected, pertaining to the dental laboratory.

